# Clinical characteristics of comorbid tic disorders in autism spectrum disorder: exploratory analysis

**DOI:** 10.1186/s13034-023-00625-8

**Published:** 2023-06-12

**Authors:** Ye Rim Kim, Da-Yea Song, Guiyoung Bong, Jae Hyun Han, Joo-Hyun Kim, Hee Jeong Yoo

**Affiliations:** 1 Department of Psychiatry, Seoul National University Bundang Hospital, 82 Gumi-Ro 173Beon-Gil, Bundang-Gu, Seongnam, 13620 Republic of Korea; 2 Department of Psychiatry, Seoul National University College of Medicine, 103 Daehak-ro, Jongno-gu, Seoul, 03080 Republic of Korea

**Keywords:** Autism spectrum disorder, Tic disorder, Yale Global Tic Severity Scale, Intelligence Quotient, Comorbidity

## Abstract

**Background:**

The frequency, clinical characteristics, and associated symptoms of comorbid tic disorders in individuals with autism spectrum disorder (ASD) remain unclear.

**Methods:**

We included subsets of individuals from a larger genetic study who were diagnosed with ASD (n = 679; age: 4–18 years) and completed the Yale Global Tic Severity Scale (YGTSS) questionnaire. Based on the YGTSS score, the individuals were divided into two groups: ASD only (n = 554) and ASD with tics (n = 125). Individuals were assessed using the verbal and non-verbal intelligence quotient (IQ), Vineland Adaptive Behavior Scale (VABS-2), Social Responsiveness Scale-2 (SRS-2), Child Behavior Checklists (CBCL), and Yale-Brown Obsessive–Compulsive Scale (YBOCS), followed by between-group comparisons. All statistical analyses were performed using the Statistical Package for the Social Sciences (SPSS) version 26.

**Results:**

Tic symptoms were observed in 125 (18.4%) participants; among them, most participants presented both motor and vocal tics (n = 40, 40.0%). The ASD with tics group had a significantly higher average age and full-scale IQ score than the ASD only group. After adjusting for age, the ASD with tics group had significantly higher scores in the SRS-2, CBCL, and YBOCS subdomains than the ASD only group. Furthermore, all variables except the non-verbal IQ and VABS-2 scores were positively correlated with the YGTSS total score. Finally, the proportion of tic symptoms was significantly higher among individuals with a higher IQ score (≥ 70).

**Conclusions:**

The IQ score was positively correlated with the proportion of tic symptoms among individuals with ASD. Moreover, the severity of the core and comorbid symptoms of ASD was associated with the occurrence and severity of tic disorders. Our findings suggest the need for appropriate clinical interventions for individuals with ASD.

*Trial registration* This study retrospectively registered participants

**Supplementary Information:**

The online version contains supplementary material available at 10.1186/s13034-023-00625-8.

## Background

Autism spectrum disorder (ASD) is primarily characterized by persistent impairment of reciprocal social communication and interactions, as well as restricted, repetitive patterns of behavior, interests, or activities (RRB). The term *spectrum* reflects the fact that the manifestations of ASD greatly vary according to the severity, functioning level, and chronological age [1]. Since ASD affects a wide range of developmental domains, including motor coordination and repetitive motor behaviors, it is a lifelong chronic neurodevelopmental disorder that affects daily functions. In addition to the core symptoms involving social communication and repetitive behaviors, individuals with ASD often present various comorbidities. Approximately 70% and 40% of individuals with ASD have at least one and two or more comorbid disorders, respectively [1]. Examples of common comorbidities in individuals with ASD include major depressive disorder, bipolar disorder, phobias, obsessive–compulsive disorder (OCD), anxiety disorder, attention deficit hyperactivity disorder (ADHD), and psychosis [2]. A recent meta-analysis indicated that the overall pooled estimated prevalence of ADHD and anxiety disorders was 28% and 20%, respectively [3].

Additionally, motor disturbances are prevalent among individuals with ASD, including gross motor coordination, fine motor coordination, motor stereotypies, postural impairment, and imitation and praxis. However, the exact mechanisms underlying these disturbances remain unclear [4]. Tics are a common motor disturbance among individuals with ASD. The severity of tic symptoms can be attenuated through several treatment strategies, including pharmacotherapy. However, the prevalence and clinical characteristics of tics in individuals with ASD remain relatively unclear compared with those of other mental disorders [5, 6]. The estimated prevalence of tics among individuals with ASD ranges from 22 to 34% depending on the sample population and administered assessment tools [5–7]. Approximately 3–20% and 3–11% of children with Tourette disorder and ASD have comorbid ASD and Tourette disorder, respectively [7–9]. Additionally, a recent study that used video-based assessment for tic evaluations by well-trained movement experts rather than the commonly used questionnaires confirmed that tics might be relatively common among young individuals with severe ASD who present heterogeneous repetitive behaviors [10]. The wide range of the reported prevalence rates of tics could be mainly attributed to the absence of standardized screening tools and the inclusion of small sample sizes.

The core clinical characteristics of comorbid disorders, including tic disorder, in individuals with ASD remain unclear. Few studies have explored the clinical characteristics of ASD in individuals with Tourette disorder. A study reported that social communication deficits in patients with Tourette disorder could reflect ASD [11]. Moreover, the developmental history of individuals with tics suggested that the observed social deficits could be attributed to ASD [11]. Co-occurrence of early-onset Tourette disorder with ASD may be a prognostic marker for positive outcomes of general developmental achievement and autistic features, which could be mediated by high Intelligence Quotient (IQ) scores in children with comorbid tics [12–14]. Accordingly, it is important to investigate the relationship between comorbid tic disorders and high IQ. Additionally, it is important to elucidate the characteristics of baseline functioning, as well as core and comorbid symptoms of ASD, in individuals with comorbid tic and Tourette disorder.

The primary aim of this study is to investigate the frequency of comorbid tic disorders in individuals with ASD in our sample using standardized assessment methods. Secondly, we aimed to explore differences in ASD symptom severity, other comorbidities, such as OCD symptoms and externalizing/internalizing behaviors, and baseline functioning through verbal/non-verbal IQ or adaptive behaviors. The hypotheses of this study are as follows: (1) clinical characteristics, including core ASD symptoms, internalizing/externalizing problems, and obsession/compulsion, are significantly higher among individuals with ASD and tics than in those without tics; (2) the severity of tic symptoms is significantly correlated with various clinical scores; (3) a high IQ score (IQ ≥ 70) is more common among individuals with ASD and comorbid tics than in individuals with ASD without tics.

## Methods

### Participants

**Fig. 1:**
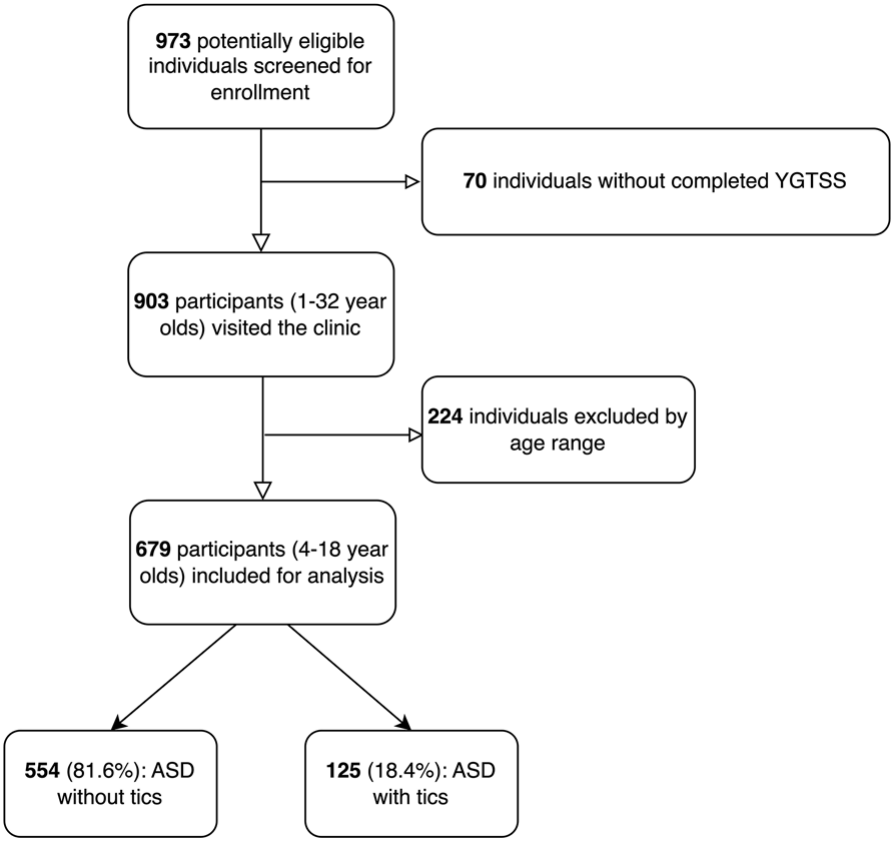
The baseline characteristics of the participants and the frequency of tic symptoms

We included individuals diagnosed with ASD and their first-degree family members pooled from a genetic study conducted at Seoul National University Bundang Hospital (SNUBH). Data were collected between September 2011 and August 2021. The inclusion criteria of probands (children with ASD identified in the genetic study) were as follows: age 4–18 years, a confirmed ASD diagnosis based on the Autism Diagnostic Observation Schedule-Second Edition (ADOS-2) [15] and the Autism Diagnostic Interview-Revised (ADI-R) [16], and parents having completed the Yale Global Tic Severity Scale (YGTSS) [17] questionnaire (Fig. 1). We excluded individuals who did not provide consent for the use of anonymized data in the retrospective analysis. Each participant had provided informed consent in the original study. The Institutional Review Board approved the retrospective analysis of the collected data (IRB no. B-2105-684-101) at SNUBH.

### Procedures

Participants were recruited from the child and adolescent psychiatric clinic at SNUBH or through advertisements posted on parent support group blogs. Informed consent was obtained from both the participants and their parents. During the first clinic visit, the Korean-translated ADOS-2 [18] and ADI-R [19], as well as verbal or non-verbal Full-Scale IQ (FSIQ) tests, were administered by trained professionals qualified to administer the ADOS-2 and ADI-R or trained graduate students under close supervision of clinical psychologists. All included participants met the threshold ADOS-2 and ADI-R scores for ASD diagnosis. Additionally, caregivers completed the Social Responsiveness Scale-Second Edition (SRS-2) questionnaire [20]. Using the aforementioned clinical information, psychiatrists confirmed the ASD diagnosis based on their clinical judgment.

Participants and caregivers who met the eligibility criteria also completed questionnaires on other neuropsychiatric symptoms, including the Child Behavior Checklist for Ages (CBCL) [21], the Korean version of the Yale-Brown Obsessive–Compulsive Scale (YBOCS) [22], and the Vineland Adaptive Behavior Scales, second edition (VABS-2) [23]. Further details regarding recruitment and screening procedures can be found in the study by Yoo et al. [24].

### Measures

#### Autism Diagnostic Observation Schedule-second edition (ADOS-2) [15, 18]

The ADOS-2 is a semi-structured ASD diagnostic instrument that is based on direct observation by a trained expert. It is comprised of five modules and is administered depending on the participant’s age and expressive language skills. Further, it is scored according to the presence or absence of behaviors and social interactions during the assessment, with a higher score suggesting severe impairment. For a confirmed autism diagnosis, the sum of the social affect and RRB domains must meet the defined threshold.

#### Autism Diagnostic Interview-Revised (ADI-R) [16, 19]

The ADI-R is a 93-item semi-structured diagnostic interview administered to caregivers, with a higher score indicating greater severity of ASD-related symptoms. The diagnostic algorithm is based on several selected items across three domains: social interaction, communication, and RRBs. Past and present mannerisms were measured using the following question items: 77, 77b, 78, and 78b, (former and latter numbers assess present and past mannerisms, respectively). The Korean-translated version of the ADI-R, which is approved by the publisher Western Psychological Services, was used [19]. The ADI-R and ADOS-2 are considered as the gold-standard diagnostic assessments; moreover, they allow even higher sensitivity and specificity when used together [25].

#### Yale Global Tic Severity Scale (YGTSS) [17, 26]

The presence, type, and severity of tics was evaluated using the parent-rated YGTSS, which is the most commonly used tool for assessing Tourette syndrome. This tool which has established reliability and validity assesses the severity of tics during the preceding week, and individuals with a YGTSS total score > 0 were classified as having tic symptoms.

The YGTSS allows the classification of motor and phonic tics across various dimensions [17]. Based on the YGTSS motor and vocal tic sub-scores, participants in the ASD with tic group (N = 125) were divided into the following groups: motor tic, vocal tic, or both.

#### WISC-IV, WIPSI-IV, and WAIS-IV

Depending on the participant’s age, FSIQ was assessed using the Korean Wechsler Intelligence Scale for Children-IV (WISC-IV), Korean Wechsler Primary and Preschool Scale Intelligence-IV (WPPSI-IV), and Korean Wechsler Adult Intelligence Scale-IV (WAIS-IV). These tools allow assessment of intelligence and cognitive ability; moreover, participants with an IQ score ≥ 70 were considered as having autism without intellectual disability.

#### Korean Leiter International Performance Scale-Revised (K-Leiter-R) [27]

Non-verbal IQ was assessed using the K-Leiter-R, which involves the provision of instructions through pantomime followed by motor-based responses. It is a well-established test that is suitable for individuals unable to complete other cognitive tests requiring vocal or expressive language skills [28–30].

#### Vineland Adaptive Behavior Scale, second edition (VABS-2) [23]

The VABS-2 is a caregiver-report questionnaire regarding adaptive behaviors. Items are rated based on how frequently or well the participant can perform them. It has four subdomains: communication, daily living skills, socialization, and motor skills. A higher total composite score across all subdomains indicates greater adaptive behavior.

#### Social Responsiveness Scale-2 (SRS-2) [20]

The parent-reported SRS-2 questionnaire was used to assess the ASD symptoms and their severity. It comprises 65 items grouped into five subscales: social awareness, social cognition, social communication, social motivation, and autistic mannerisms. A higher score indicates greater severity of ASD symptoms. The SRS-2 has good internal consistency and concurrent discriminant validity. The present study used the standardized total SRS-2 T-score as well as the subscale scores for social communication impairments and autistic mannerisms.

#### Child Behavior Checklist (CBCL) [21, 31]

The CBCL is a parent-rated scale for evaluating behavioral issues in children during the past 6 months. Participants aged 4–5 years and 6–18 years were assessed using the CBCL versions for 1.5–5- and 6–18-year-olds, respectively [31].

The CBCL has good validity and reliability [21]. It includes several domains, such as emotional reactivity, anxiety/depression, somatic complaints, withdrawal, sleep problems, attention problems, and aggressive behavior. Scores for internalizing, externalizing, and total problems were obtained, with the internalizing domain reflecting a broad assessment of emotional problems, including anxiety and depression symptoms, while the externalizing domain assesses behavioral problems such as rule-breaking behavior and aggressive behavior syndrome [32]. This study included sub-scores and total scores for both internalizing and externalizing problems.

#### Yale-Brown Obsessive–Compulsive Scale (YBOCS) [22]

The YBOCS is a parent-reported, 10-item questionnaire used for quantitative assessment of OCD symptoms. We included total and sub-scores for analysis and relied on parental reports for data collection.

### Statistical analyses

Baseline normality tests were conducted, and between-group comparisons of ASD symptoms, other neuropsychiatric symptoms, and baseline characteristics were performed. Given the non-normal distribution of continuous variables, the Mann–Whitney U test was used for between-group comparisons. Quade’s rank of covariance was used to correct the effect of age differences [33].

Pearson’s correlation was used to explore the association of tic severity with ASD symptoms, other neuropsychiatric symptoms, and baseline characteristics. The chi-square test was used to assess the relationship between autism without intellectual disability and the presence of comorbid ASD symptoms. Descriptive statistics and correlation analyses were conducted using SPSS version 26 (IBM Corp.), with statistical significance set at *p* < 0.05.

The sample size was calculated using G*power 3.1.9.7, based on a similar study with an effect size of 0.32 [29]. Setting effect sizes at 0.32, the sample sizes for Groups 1 and 2 (ASD without tics vs. ASD with tics) were 593 and 137, respectively (α = 0.05, 1-β (power) = 0.95), which was met by our pooled sample. Effect sizes were calculated through a sensitivity power analysis using G*power 3.1.9.7 [34].

## Results

### Patient characteristics

#### Type of tics

In the ASD with tics group (N = 125, 18.4%), there were 44 (35.2%), 31 (24.8%), and 50 (40.0%) participants with motor tics, vocal tics, and both, respectively. There were no significant between-group differences in sex, non-verbal intelligence scores, and adaptive behaviors. However, the mean age (ASD with tic: 8.02 years, ASD only: 6.68 years) and FSIQ scores (ASD with tic: 95.37, ASD only: 81.46) were significantly higher in the ASD with tic group than in the ASD only group (*p* < 0.001). Moreover, the VABS-2 total score was significantly higher in the ASD only group than in the ASD with tics group after adjusting for the effect of age (ASD with tic: 67.47, ASD only: 69.75) (*p* = 0.003). Table 1 shows the characteristics of both groups.

**Table 1:** Baseline characteristics of both groups

	Total	ASD only	ASD + tic	p-value^†^	p-value^‡^
Age, years	6.93 $\pm$ 3.50	6.68 $\pm$ 3.37	8.02 $\pm$ 3.83	< 0.001**	
Male (%)	571 (84.1%)	463 (83.6%)	108 (86.4%)	0.519	
FSIQ	84.38 $\pm$ 23.84	81.46 $\pm$ 24.03	95.37 $\pm$ 19.76	< 0.001**	0.015*
K-Leiter-R	74.01 $\pm 25.21$	73.23 $\pm$ 24.77	78.56 $\pm$ 27.41	0.087	0.233
VABS-2	69.34 $\pm 30.75$	69.75 $\pm$ 30.68	67.47 $\pm$ 31.17	0.056	0.003*

ASD: autism spectrum disorder; FSIQ: full-scale intelligence quotient; VABS-2: Vineland Adaptive Behavior Scales; K-Leiter-R: Korean Leiter International Performance Scale-Revised

Continuous and categorical variables are expressed as mean ± standard deviation and n (%), respectively

*p < 0.05, **p < 0.001

^†^Mann–Whitney U test

^‡^Quede’s rank of covariance, adjusted for age

### Between-group comparison of clinical characteristics according to the presence of tic symptoms

Compared with the ASD only group, the ASD with tic group showed significantly higher scores for the SRS-2 sub-scales, emotional and behavioral symptoms in the CBCL, and OCD symptoms (*p* < 0.001). This trend remained even after adjusting for age (Table 2).

**Table 2:** Between-group comparison of clinical characteristics according to the presence of tic symptoms

	Total	ASD only	ASD + tic	p-value^†^	p-value^‡^
SRS-2 SCI T score	74.11 $\pm$ 11.04	73.21 $\pm$ 10.97	78.02 $\pm$ 10.51	< 0.001**	< 0.001**
SRS-2 RRB T score	68.41 $\pm$ 11.89	67.10 $\pm$ 11.59	74.16 $\pm$ 11.54	< 0.001**	< 0.001**
SRS-2 total T score	73.49 $\pm$ 11.10	72.48 $\pm$ 10.98	77.89 $\pm$ 10.55	< 0.001**	< 0.001**
CBCL anxiety/depression	59.11 $\pm$ 9.25	58.14 $\pm 8.74$	63.35 $\pm 10.20$	< 0.001**	< 0.001**
CBCL withdrawn/depression	65.06 $\pm$ 9.10	64.39 $\pm$ 8.68	67.94 $\pm$ 10.26	< 0.001**	< 0.001**
CBCL somatic complaints	55.30 $\pm 6.78$	54.61 $\pm$ 6.35	58.26 $\pm$ 7.77	< 0.001**	< 0.001**
CBCL delinquent behaviors	61.05 $\pm$ 6.26	60.51 $\pm$ 6.22	62.97 $\pm$ 6.05	0.001*	0.001*
CBCL aggressive behaviors	59.99 $\pm$ 8.85	59.06 $\pm$ 8.49	64.05 $\pm$ 9.25	< 0.001**	< 0.001**
CBCL internalizing behaviors	60.85 $\pm$ 10.41	59.63 $\pm$ 9.92	66.16 $\pm$ 10.78	< 0.001**	< 0.001**
CBCL externalizing behaviors	60.89 $\pm$ 10.63	59.87 $\pm$ 10.31	65.36 $\pm$ 10.92	< 0.001**	< 0.001**
YBOCS obsession	3.22 $\pm$ 4.52	2.85 $\pm$ 4.16	4.97 $\pm$ 5.63	< 0.001**	< 0.001**
YBOCS compulsion	3.17 $\pm$ 4.45	2.78 $\pm$ 4.04	5.00 $\pm$ 5.70	< 0.001**	< 0.001**
YBOCS total	6.38 $\pm$ 8.71	5.62 $\pm$ 7.97	9.97 $\pm$ 10.95	< 0.001**	< 0.001**

ASD: autism spectrum disorder; SRS-2: Social Responsiveness Scale; SCI: Social Communication Index; RRB: Restricted Interests and Repetitive Behavior; CBCL: Child Behavior Checklist; YBOCS: Yale-Brown Obsessive Compulsive Scale

*p < 0.05, **p < 0.001

^†^Mann–Whitney U test

^‡^Quede’s rank of covariance, adjusted for age

### Correlation of tic severity with clinical variables

Except for the non-verbal IQ (K-Leiter-R) and VABS-2 total scores, all demographic and clinical variables showed a clinically significant positive correlation (*p* < 0.001; except for FSIQ, *p* = 0.034) with the YGTSS total score (Table 3).

**Table 3:** Correlation of tic severity with clinical variables

	Pearson coefficient	p-value	N
Age, years	0.154	< 0.001**	679
FSIQ	0.152	0.034*	195
K-Leiter-R	0.028	0.586	369
VABS-2	− 0.066	0.096	632
SRS-2 SCI T score	0.173	< 0.001**	663
SRS-2 RRB T score	0.248	< 0.001**	668
SRS-2 total T score	0.194	< 0.001**	663
CBCL anxiety/depression	0.206	< 0.001**	667
CBCL withdrawn/depression	0.139	< 0.001**	663
CBCL somatic complaints	0.202	< 0.001**	661
CBCL delinquent behaviors	0.157	0.001*	478
CBCL aggressive behaviors	0.236	< 0.001**	667
CBCL internalizing behaviors	0.224	< 0.001**	667
CBCL externalizing behaviors	0.199	< 0.001**	667
YBOCS obsession	0.178	< 0.001**	609
YBOCS compulsion	0.200	< 0.001**	607
YBOCS total	0.195	< 0.001**	609

FSIQ: full-scale intelligence quotient; VABS-2: Vineland Adaptive Behavior Scales; SRS-2: Social Responsiveness Scale; CI: Social Communication Index; RRB: Restricted Interests and Repetitive Behavior; CBCL: Child Behavior Checklist; YBOCS: Yale-Brown Obsessive Compulsive Scale

*p < 0.05, **p < 0.001

### Relationship between autism without intellectual disability (IQ ≥ 70) and comorbid tic disorders

Participants with an IQ score ≥ 70 had a significantly higher proportion of comorbid tic symptoms than those with an IQ < 70 (odds ratio = 3.93, 95% confidence interval: 1.56–9.90 [p = 0.001]) (Table 4).

**Table 4:** Relationship between autism without intellectual disability and comorbid tic disorders

	IQ < 70	IQ ≥ 70	Total	Odds ratio (95% confidence interval)	p-value
ASD only	62 (91.2%)	92 (72.4%)	154 (79.0%)	3.931 (1.560–9.904)	0.001*
ASD + tic	6 (8.8%)	35 (27.6%)	41 (21.0%)		
Total	68 (100%)	127 (100%)	195 (100%)		

ASD: autism spectrum disorder; IQ: Intelligence Quotient

*p < 0.05, **p < 0.001

## Discussion

In our study, the prevalence of tic symptoms in individuals with ASD (18.4%) was higher than that in the general population (0.77–2.99%) [35]. Further, the prevalence of tic symptoms in individuals with ASD (18.4%) was higher than the reported prevalence of Tourette disorder in individuals with ASD (3–11%) [7–9]. The previously reported prevalence of tics among individuals with ASD ranges from 22 to 34% [6, 7]. The wide range of the reported prevalence rates of tics could be mainly attributed to the absence of standardized screening tools and the inclusion of small sample sizes. Therefore, the findings should be interpreted with caution. Based on the findings of previous studies [6, 36, 37], chronic motor tics are more common than chronic vocal tics in various groups; moreover, the prevalence of comorbid vocal and motor tics is higher or similar to that of only chronic motor tics. Consistent with findings of previous studies, the co-occurrence of motor and vocal tics was the most frequent, while the occurrence of only vocal tics was the least frequent.

Notably, the FSIQ was positively correlated with tic severity, which could be attributed to the fact that tics might be easier to differentiate in children with higher IQ scores. The presence of tics in individuals with early-life ASD may be a positive prognostic factor [13], with this relationship being mediated by the generally higher IQ levels in these individuals [38]. In our study, individuals without intellectual disability showed a higher rate of comorbid tics than individuals with intellectual disability. However, the exact mechanism or causal relationships of IQ with tic symptoms in individuals with ASD remains unclear; therefore, further research is warranted. Our findings are inconsistent with the general notion that patients with tic disorders without ASD have a lower IQ than the general population [39, 40]. Contrastingly, our findings are consistent with a previous report that tics and Tourette’s syndrome are extremely common in individuals with autism, especially those with a high IQ [41]. Further, our findings suggest that comorbid ASD should be suspected in patients with both chronic tic symptoms and social deficits, as well as a normal IQ. Contrastingly, regarding adaptive behaviors, the standardized VABS-2 score was higher in the ASD only group than in the ASD with tics group.

We observed significant between-group differences in all core and comorbid symptoms of ASD, with the ASD with tics group showing higher scores. Furthermore, tic severity showed a significant positive correlation with several clinical variables. These findings could be attributed to the fact that tic symptoms are considered as RRB symptoms on various scales measuring ASD core symptoms. Furthermore, due to mediating factors such as social deficits, the severity of ASD core symptoms could be worse than that of ASD alone [42]. Notably, the ASD with tics group tended to have more past and current mannerisms detected using ADI-R subscales than the ASD only group, which could be mediated by RRB symptoms in individuals with ASD. However, there was no significant between-group difference. Therefore, further studies are warranted to elucidate the relationships between motor disturbances. Additionally, prospective studies on the trajectory of tics and mannerisms in individuals from an early age are warranted. Moreover, cognitive deficits in executive functioning and attention may impact ASD core symptoms. Regarding comorbid conditions, individuals with both tics and ASD showed multiple behavioral and emotional comorbidities, which may have synergistic effects on each other. Future studies are warranted to investigate mediating factors the relationship between tic symptoms and social difficulties.

The strengths of this study include the use of a large-scale sample recruited from a single center and the use of standardized tests. To our knowledge, this is the first study to compare comorbid conditions between patients with ASD with and without tics. This study has a few limitations. First, there may be a possible confounding effect of age, which we adjusted for using Quade’s rank of covariance. Second, we did not consider previous medications. However, we analyzed current medications according to specific psychiatric medication groups (See Additional file 1: Table S1, Additional file 2: Table S2, Additional file 3: Table S3 for more details). Third, we only used the parent-reported YGTSS to assess the severity of tics; however, it has shown good discriminative ability [43]. Moreover, the YGTSS could have a limited ability in discriminating tic symptoms from ASD stereotypes and compulsive behavior in OCD. However, we observed a significant correlation between the parent-reported “present tics” and positive YGTSS scores. Additionally, there was a low proportion of participants with a positive YGTSS score and mannerisms or rituals on the ADI-R. Therefore, it is unlikely that parents misinterpreted tics as compulsive behaviors or mannerisms. Fourth, this was a retrospective study, which is limited by the time-sensitive nature of the data. Fifth, our findings could have been influenced by the presence of other comorbidities; however, we analyzed several other comorbidities, including OCD, internalizing symptoms, and externalizing symptoms. Taken together, individuals with comorbid ASD and tics may be more clinically susceptible because they are usually accompanied by other comorbidities. Therefore, professionals should identify and manage individuals with comorbid ASD and tics. Finally, we did not consider the history and severity of tics since they were only assessed using the YGTSS.

## Conclusions

A high IQ (≥ 70) was significantly associated with tic disorders in individuals with ASD. Moreover, the severity of ASD core and comorbid symptoms was significantly associated with tic symptoms. Our findings suggest that comorbid tic disorders are more common in children with ASD than in the general population. Therefore, close surveillance for comorbid tic disorders in individuals with ASD, especially those without intellectual disabilities, is important.

## Supplementary Information

**Additional file 1: Table S1**. Number of participants using current psychiatric medications.

**Additional file 2: Table S2.** Number of participants using specific psychiatric medications.

**Additional file 3: Table S3.** Relationship between tics identified using the YGTSS and current psychiatric medications.

## Data Availability

The datasets generated during and/or analyzed during the current study are not publicly available as the IRB approved the data to be used within the research team but could be available from the corresponding author on reasonable request.

## Abbreviations

ASD: Autism Spectrum Disorder
OCD: Obsessive–compulsive disorder
ADHD: Attention-deficit hyperactivity disorder
IQ: Intelligence Quotient
SNUBH: Seoul National University Bundang Hospital
ADOS-2: Autism Diagnostic Observation Schedule-Second Edition
ADI-R: Autism Diagnostic Interview-Revised
YGTSS: Yale Global Tic Severity Scale
FSIQ: Full-scale IQ
SRS-2: Social Responsiveness Scale-Second Edition
CBCL: Child Behavior Checklist for Ages
YBOCS: Yale-Brown Obsessive Compulsive Scale
K-Leiter-R: Korean Leiter International Performance Scale-Revised
VABS-2: Vineland Adaptive Behavior Scales, second edition
WISC-IV: Wechsler Intelligence Scale for Children-IV
WPPSI-IV: Wechsler Primary and Preschool Scale Intelligence-IV
WAIS-IV: Wechsler Adult Intelligence Scale-IV
SCI: Social Communication Index
RRB: Restricted Interests and Repetitive Behavior
