# Supplementary material for: Clinical characteristics of comorbid tic disorders in autism spectrum disorder: exploratory analysis
Source: Child Adolesc Psychiatry Ment Health. 2023 Jun 12;17:71. doi: 10.1186/s13034-023-00625-8 (PMC10262579; doi:10.1186/s13034-023-00625-8)
Supplement: Supplementary file 1 — Additional file 1: Table S1. Number of participants using current psychiatric medications. [file 13034_2023_625_MOESM1_ESM.docx]

**Table S1.** Number of participants using current psychiatric medications

|  | No current medication | Current medications | Total | p-value |
| --- | --- | --- | --- | --- |
| ASD only | 418 (81.5%) | 136 (81.9%) | 554 (81.6%) | 0.897 |
| ASD + tic | 95 (18.5%) | 30 (18.1%) | 125 (18.4%) |  |
| Total | 513 (100%) | 166 (100%) | 679 (100%) |  |

ASD, autism spectrum disorder

* *p* < 0.05, ** *p* < 0.001
