# Supplementary material for: Clinical characteristics of comorbid tic disorders in autism spectrum disorder: exploratory analysis
Source: Child Adolesc Psychiatry Ment Health. 2023 Jun 12;17:71. doi: 10.1186/s13034-023-00625-8 (PMC10262579; doi:10.1186/s13034-023-00625-8)
Supplement: Supplementary file 2 — Additional file 2: Table S2. Number of participants using specific psychiatric medications. [file 13034_2023_625_MOESM2_ESM.docx]

**Table S2.** Number of participants using specific psychiatric medications

|  | Number of participants currently taking stimulants | Number of participants currently taking antipsychotics | Number of participants currently taking antidepressants | Number of participants currently taking sedatives | Number of participants currently taking mood stabilizers | Number of participants currently taking psychiatric medication |
| --- | --- | --- | --- | --- | --- | --- |
| ASD without tics | 61 | 90 | 36 | 9 | 13 | 136 |
| ASD with tics | 17 | 24 | 6 | 3 | 2 | 30 |
| Total | 78 | 114 | 42 | 12 | 15 | 166 |
