# Supplementary material for: Clinical characteristics of comorbid tic disorders in autism spectrum disorder: exploratory analysis
Source: Child Adolesc Psychiatry Ment Health. 2023 Jun 12;17:71. doi: 10.1186/s13034-023-00625-8 (PMC10262579; doi:10.1186/s13034-023-00625-8)
Supplement: Supplementary file 3 — Additional file 3: Table S3. Relationship between tics identified using the YGTSS and current psychiatric medications. [file 13034_2023_625_MOESM3_ESM.docx]

**Table S3.** Relationship between tics identified using the YGTSS and current psychiatric medications

|  |  | **ASD only** | **ASD + Tic** | **Total** | **p-value** |
| --- | --- | --- | --- | --- | --- |
| Stimulant | No | 75 (85.2%) | 13 (14.8%) | 88 (100%) | ​​0.241 |
|  | Yes | 61 (78.2%) | 17 (21.8%) | 78 (100%) |  |
| Antipsychotics | No | 46 (88.5%) | 6 (11.5%) | 52 (100%) | 0.140 |
|  | Yes | 90 (78.9%) | 24 (21.1%) | 114 (100%) |  |
| Antidepressants | No | 100 (80.6%) | 24 (19.4%) | 124 (100%) | 0.461 |
|  | Yes | 36 (85.7%) | 6 (14.3%) | 42 (100%) |  |
| Sedatives | No | 127 (82.5%) | 27 (17.5%) | 154 (100%) | 0.456 |
|  | Yes | 9 (75.0%) | 3 (25.0%) | 12 (100%) |  |
| Mood stabilizers | No | 123 (81.5%) | 28 (18.5%) | 151 (100%) | 1.000 |
|  | Yes | 13 (86.7%) | 2 (13.3%) | 15 (100%) |  |
